# Sedative-hypnotic properties and mitochondrial effects of coenzyme Q2 in mice

**DOI:** 10.1016/j.cmp.2025.12.001

**Published:** 2025-12-19

**Authors:** Haeun Lim, Rong Lu, Sahdev N. Patel, Sebastian Zinn, Paul S. García, Richard J. Levy

**Affiliations:** a Department of Anesthesiology, Columbia University Irving Medical Center, NY 10032, USA; b Goethe University Frankfurt, University Hospital, Department of Anesthesiology, Intensive Care Medicine and Pain Therapy, Frankfurt am Main 60590, Germany

**Keywords:** Anesthesia, Mitochondria, Benzoquinone, Coenzyme Q, Membrane potential, Proton leak

## Abstract

**Background:**

Anesthetics are fascinating drugs capable of inducing reversible unconsciousness. Functional targets of these agents exist within mitochondria and disruption of bioenergetic capacity plays a role in mediating the anesthetic response. Recently, ubiquinone-5 (coenzyme Q1)(Ub5 or CoQ1), a short-chain coenzyme Q (CoQ) analog with a single isoprene unit, was identified as a novel anesthetic with a mitochondrial mechanism. Benzoquinones share an identical head group yet differ in the length of their isoprenoid tail. It is unknown if other CoQ analogs exhibit anesthetic properties or how side-chain length affects their activity. Here, we hypothesized that CoQ2, an analog with two isoprene tail units, would act as a sedative-hypnotic and that its mitochondrial biological activity would differ from Ub5 (CoQ1).

**Methods:**

Behavioral phenotype was assessed in mice using behavioral and neurophysiological approaches. We measured activity within isolated mitochondria using polarography and spectrophotometry and attempted to identify source of proton leak using pharmacological inhibitors.

**Results:**

CoQ2 induced brief and reversible brain quiescence shortly after injection and caused a precipitous decline in the mitochondrial membrane potential due to excessive mitochondrial proton leak combined with electron transport chain inhibition. Unlike Ub5 (CoQ1), however, there was a latency to onset of CoQ2-induced hypnosis, effective CoQ2 doses were relatively higher, and duration of CoQ2-mediated unconsciousness was relatively longer than that previously reported for Ub5 (CoQ1). Furthermore, the source of proton leak differed between analogs.

**Conclusions:**

The comparable anesthetic effects likely relate to the benzoquinone head group shared between molecules while the disparities are likely due to the length of the isoprenoid tail.

## Introduction

Anesthetics are an extraordinary class of drugs capable of inducing reversible unconsciousness, amnesia, and immobilization [1]. They are promiscuous agents with a variety of putative cellular targets and mechanisms [1]. Interest in the mitochondrion as an effector of anesthesia-induced unconsciousness has accelerated over the last few decades and specific mitochondrial targets have been identified [2–10]. The best example of such a target is Complex I of the electron transport chain (ETC) [4–10]. Loss-of-function studies linking genetic mutations of Complex I with anesthetic hypersensitivity in various species across evolutionary biology have elucidated a role that mitochondria play in mediating the anesthetic response [5,6,9,10]. From a mechanistic standpoint, anesthetic-mediated Complex I inhibition is thought to silence synapses by disrupting bioenergy availability required for presynaptic neuronal endocytosis and neurotransmitter recycling [4]. However, the exact details are not fully understood.

Recently, we identified a novel anesthetic agent with a discrete mitochondrial mechanism of action [11]. Coenzyme Q1 (CoQ1) or ubiquinone-5 (Ub5) (2,3-Dimethoxy-5-methyl-6-(3-methyl-2-butenyl)-1,4-benzoquinone, CAS Registry Number 727–81–1), a synthetic short-chain coenzyme Q (CoQ) analog with a single isoprene tail unit, caused mice to immediately lose their righting reflex upon intravenous injection [11]. The short side-chain permitted Ub5 (CoQ1) to rapidly diffuse across membranes to penetrate the central nervous system (CNS) [12]. Loss of wakefulness resulted from a precipitous decline in the mitochondrial membrane potential (ΔΨm) caused by Ub5 (CoQ1)-induced mitochondrial proton leak and ETC inhibition [11].

There are several synthetic and naturally occurring 1,4-benzoquinones in the quinone class of compounds. All CoQ analogs share an identical benzoquinone head, however, differ in the length of their isoprenoid tail [13]. Longer side-chains render analogs relatively more hydrophobic than their shorter chain relatives and alter quinone biological activity within mitochondria and the cell [13]. However, it is unknown if side-chain length affects benzoquinone anesthetic activity. In the current work, we hypothesized that other CoQ analogs would exhibit sedative-hypnotic properties and that a longer isoprenoid tail would alter the mechanism of action. We tested our hypothesis in mice injected via tail vein with CoQ2 (2,3-Dimethoxy-5-methyl-6-geranyl-1,4-benzoquinone, CAS Registry Number 606–06–4); a synthetic quinone analog with two isoprene tail units. The findings presented here advance our understanding of the benzoquinone sub-class of anesthetics and provide insight into how isoprenoid tail length may modify their mechanism(s) of action and behavioral effects.

## Materials and methods

### Animals

The care of mice in this work was in accordance with NIH and Columbia University Irving Medical Center Institutional Animal Care and Use Committee and ARRIVE guidelines and conformed to the provisions of the Animal Welfare Act (NIH/DHHS) and the Association for Assessment and Accreditation of Laboratory Animal Care (AAALAC). C57Bl/6 N male mice (6–8 weeks old, 20–25 grams) were acquired (Charles River, Wilmington MA) and utilized for all experiments except when otherwise stated. Mice were housed in a temperature-controlled environment (maintained at 22 ± 2°C) with a 12-hour light/dark cycle and ad libitum access to food and water.

### Aralar knockout mice

Mice with a heterozygotic mutation of the *Aralar* (*Slc25a12*) gene on a SVJ129 x C57BL/6 background were acquired (Taconic Biosciences, Germantown, NY) and mated to yield *Aralar*^*−/−*^ mice and wild-type (*Aralar*^*+/+*^) littermate controls. Genotype was determined by standard PCR. *Aralar* knockout mice and wild-type controls were studied at 10 days of life given the progressive neurologic impairment and shortened lifespan in mutants [14].

## Experimental design

### Coenzyme Q2 injection

Awake mice were randomly injected via tail vein with a single dose of coenzyme Q2 (20 mg/mL in intralipid).

### Righting reflex assessment

Righting reflex was tested after tail vein injection for each mouse. Mice had an intact righting reflex if they successfully returned to prone position twice in a row [15]. Otherwise, they were considered to have lost the righting reflex. Latency to onset of loss of righting and return of righting were recorded.

### Electroencephalogram acquisition

The OpenBCI Cyton board (OpenBCI, NY, USA) was used with subdermal needle electrodes (Rhythmlink, SC, USA) to extract single-channel electroencephalogram (EEG) features, as described [16]. Reference and ground electrodes were attached as clips, one to each ear. Data were recorded at 250 Hz using the OpenBCI^™^ graphical user interface (v6.0.0-beta.1, 2023–9–28, 64-BIT, Windows 10) and saved as a.txt file. MATLAB R2024b (The Mathworks, Natick, MA, USA) for preprocessing and plotting. The signal was filtered between.5–45 Hz using a Butterworth MATLAB-based zero-phase shift routine (filtfilt).

### Mitochondrial isolation

Forebrain was harvested, homogenized in ice-cold isolation buffer (225 mM mannitol, 75 mM sucrose, 1 mM EGTA, 5 mM HEPES-KOH (pH 7.2) and 1 mg/mL of fatty-acid-free bovine serum albumin (BSA)), and centrifuged (1100 *g*) for 5 min at 4 °C. Supernatant was mixed with 80 vol% Percoll solution and layered on 10 % Percoll solution and then spun (18,500 *g* for 10 min at 4 °C). The pellet was resuspended in 250 mM sucrose, 5 mM HEPES-KOH (pH 7.2), 0.1 mM EGTA and 1 mg/ mL of BSA and centrifuged (10,000 *g* for 5 min at 4 °C). Mitochondrial pellet was resuspended and protein concentrations determined using the Lowry method.

### Mitochondrial oxygen consumption

Forebrain mitochondria (0.2 mg) were added to 1 mL of respiration buffer (200 mM sucrose, 25 mM KCl, 2 mM K2HPO4, 5 mM HEPES-KOH (pH 7.2), 5 mM MgCl2, 0.2 mg/mL BSA). Oxygen consumption was measured using a Clark-type electrode (Oxytherm, Hansatech, UK) with Complex I-dependent substrates (10 mM glutamate and 5 mM malate) or Complex II-dependent substrate (10 mM succinate in the presence of 5 μM rotenone) at 32 °C. ADP (150 μM) initiated state 3 respiration. Oligomycin (2.5 μg/mL) induced state 4 respiration and dinitrophenol (DNP) (70 μM) induced maximal rate of uncoupled state 3 respiration.

For leak respiration, oxygen consumption and ΔΨm were simultaneously measured with 5 mM succinate using forebrain mitochondria (0.2 mg) in 1-mL of respiration buffer (200 mM sucrose, 25 mM KCl, 2 mM K2HPO4, 5 mM HEPES-KOH (pH 7.2), 5 mM MgCl2, 0.2 mg/mL BSA) containing 80 ng/mL nigericin (to collapse ΔpH), 5 μM rotenone, and oligomycin (2.5 μg/mL) at 37 °C. ΔΨm was quantified using an selective ion sensitive electrode for tetraphenylphosphonium (TPP^+^) (World Precision Instruments, Sarasota, FL) and calculated using the Nernst equation [17].

In separate experiments, cyclosporine A (1 μM), carboxyatractylo-side (1 μM), guanosine diphosphate (0.75 mM), and pyridoxal 5’-phosphate (200 μM) were added to inhibit the mitochondrial permeability transition pore, the adenine nucleotide translocase, uncoupling proteins, and Aralar, respectively, to determine source of leak [18–22]. p-hydroxymercuribenzoate (10 μM) was added as a non-specific leak inhibitor [23–29]. Equal volume water served as a control vehicle.

### Electron transport chain enzyme steady-state activities

Inhibitor sensitive ETC complex activity was measured in 1-mL using spectrophotometry [30,31]. Rotenone-sensitive Complex I specific activity was measured in isolated mitochondria (40 μg) using 4.8 mM^−1^ cm^−1^ as the extinction coefficient of NADH at 340 nm with a reference wavelength of 380 nm. 2-Thenoyltrifluoroacetone-sensitive Complex II activity was measured in mitochondria (40 μg) using 19.1 mM^−1^ cm^−1^ as the extinction coefficient of 2,6-dichlorophenolindophenol at 600 nm. For Complexes III and IV, inhibitor-sensitive first-order rate constants were calculated using 18.5 mM^−1^ cm^−1^ as the extinction coefficient of cytochrome c at 550 nm in 4 μg and 2 μg mitochondria, respectively. Oligomycin-sensitive Complex V specific activity was measured with 6.2 mM^−1^ cm^−1^ as the extinction coefficient of NADH at 340 nm in mitochondria (40 μg). Rotenone-sensitive Complex I+III linked activity and anti-mycin A-sensitive Complex II+III linked activity were measured in mitochondria (40 μg) using 18.5 mM^−1^ cm^−1^ as the extinction coefficient of cytochrome c at 550 nm.

## Statistical analysis

Statistical analysis was performed with GraphPad Prism 10 software (GraphPad Software, La Jolla, CA). Data are presented in the figures as means ± SD (unless otherwise specified in the figure legends). The sample number of mice or replicates (*n*) studied for each outcome is indicated for each figure. Sample size was chosen *a priori* for each outcome measure to provide 80 % power to detect an effect size of 15 % between groups at a significance level of 0.05. Statistical tests utilized are detailed in each figure legend. Student’s t test (two-tailed) was used to determine significance between two groups and one-way analysis of variance (ANOVA) with Tukey’s post hoc test was used to calculate significance between more than two groups. Regression analysis was utilized to assess dose-dependent effect. Significance was set at p < 0.05.

## Results

### Sedative-hypnotic properties of coenzyme Q2

**Fig. 1.**
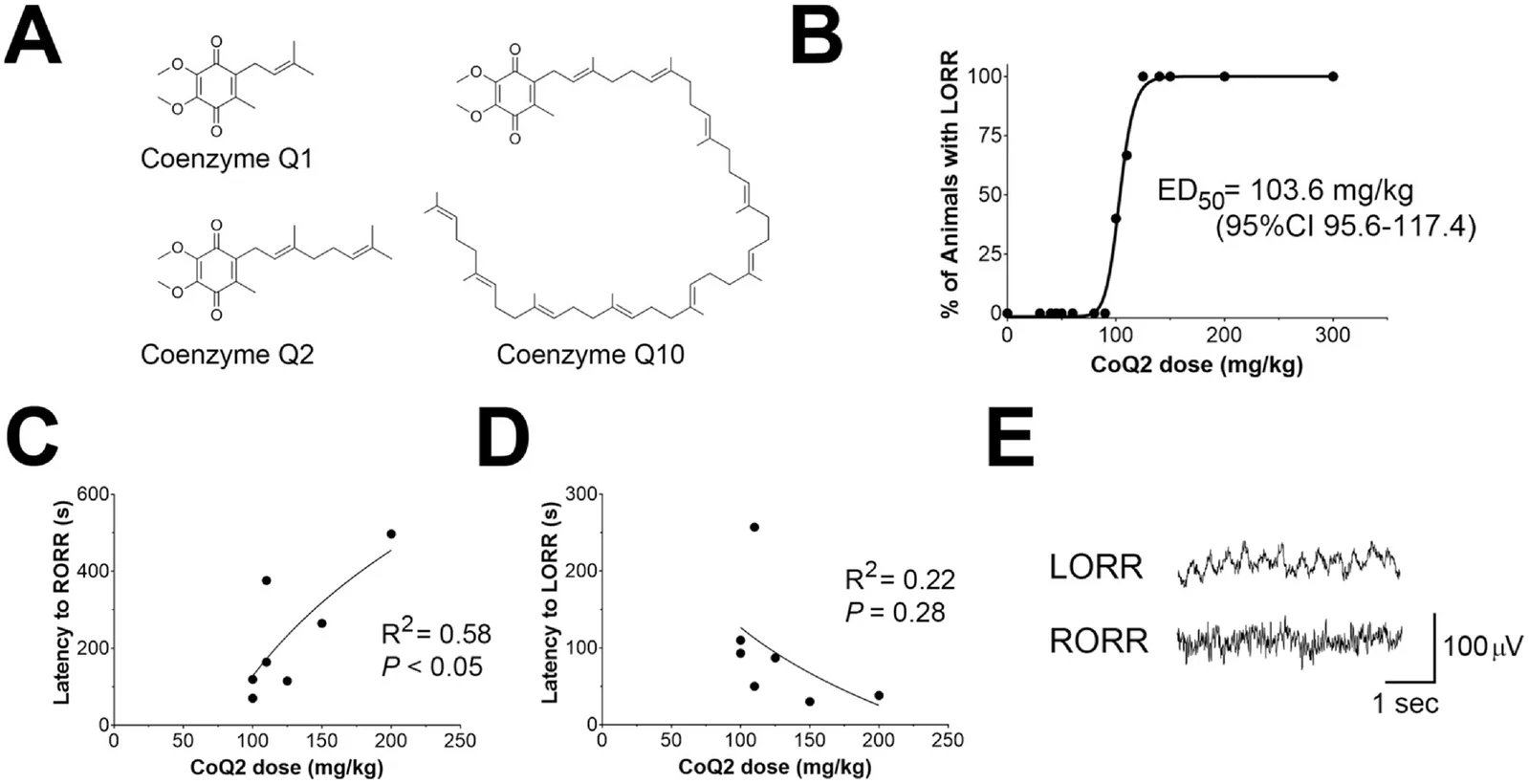
Anesthetic properties of coenzyme Q2. (A) Structure of coenzyme Q10 and its synthetic analogs, coenzyme Q1 (ubiquinone-5) and coenzyme Q2. (B) Loss of righting reflex (LORR) dose-response. (C) Latency to return of righting reflex (RORR). (D) Latency to LORR. (E) Representative electroencephalogram (EEG) traces during LORR and RORR in a mouse injected with CoQ2 (200 mg/kg). Regression analysis was applied to (B-D). *n* = 35 for (B) and *n* = 7 for (C, D).

The side-chain of CoQ2 contains one more isoprene tail unit than Ub5 (CoQ1) (Fig. 1A). It is unknown how this longer side-chain affects benzoquinone anesthetic activity. So, first, we tested the hypothesis that CoQ2 could interrupt wakefulness by injecting mice with various doses via tail vein. We then determined latency to onset of loss of the righting reflex (LORR) and duration until return of the righting reflex (RORR). As with Ub5 (CoQ1), CoQ2 caused short-lived LORR over a range of doses (Fig. 1B) [11]. Mice regained their righting reflex within ~8 min and latency to RORR demonstrated a significant and positive correlation with CoQ2 dose (p < 0.05) (Fig. 1C). Unlike Ub5 (CoQ1), though, there was a delay in the onset of CoQ2-induced LORR following injection in all animals (Fig. 1D). The mean duration of latency to LORR was 95.0 s (95 % CI: 23.3–166.7) across CoQ2 doses (Fig. 1D). However, the correlation between latency to onset of LORR and CoQ2 dose was weak and lacked significance, indicating a dose-independent relationship (Fig. 1D). We then assessed the murine electroencephalogram (EEG) upon LORR and following RORR after injection of 200 mg/kg CoQ2. Slow waves in the delta range (1–4 Hz) were prominent during loss of righting while higher frequency activity was seen upon return of righting (Fig. 1E). The slow-wave EEG activity during LORR indicated CoQ2-induced change in cortical activity comparable to a hypnotic state. Thus, CoQ2 exhibits sedative-hypnotic properties and could be considered an anesthetic.

### Coenzyme Q2 compromises ΔΨm in forebrain mitochondria

**Fig. 2.**
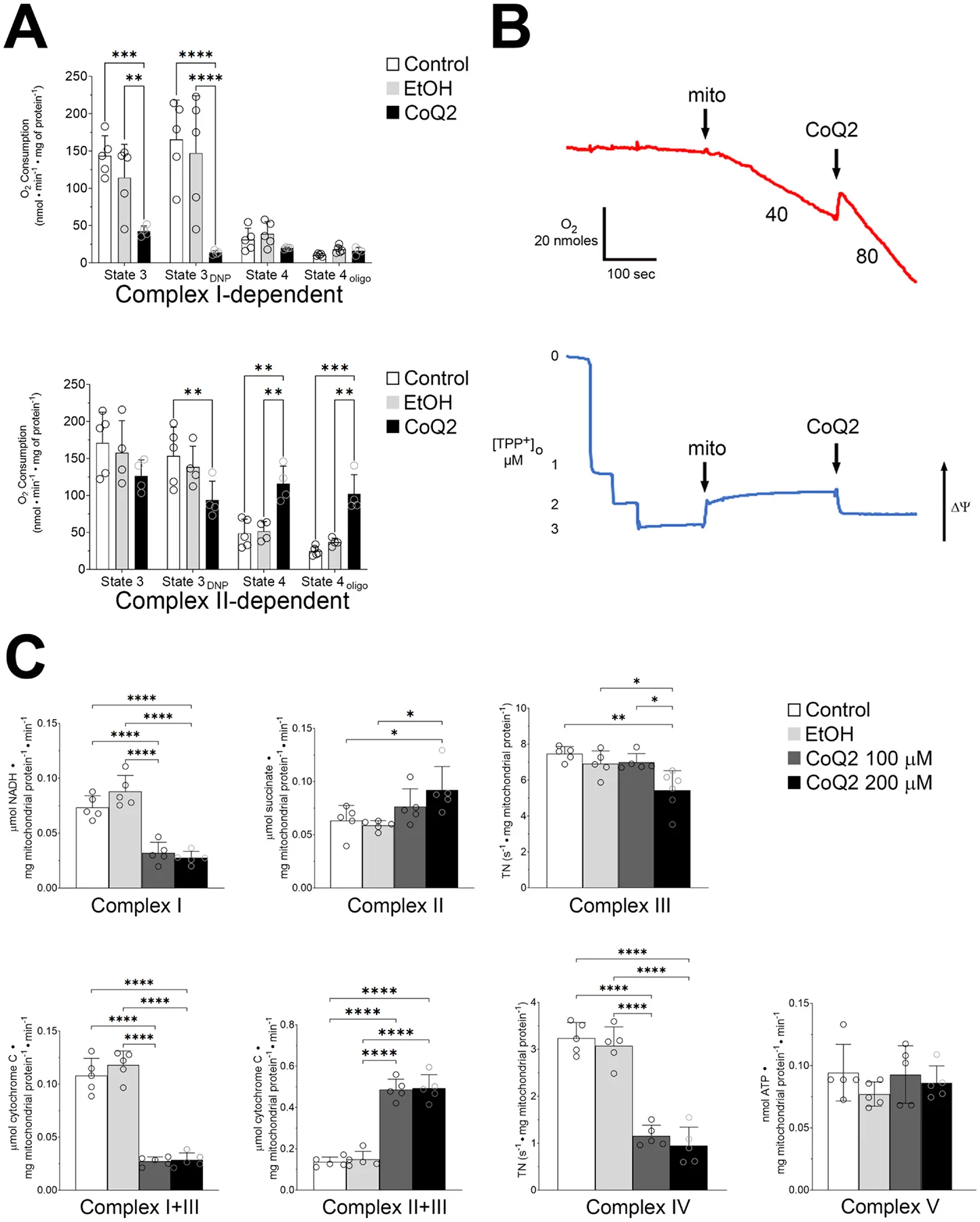
Coenzyme Q2 induces excessive and uncompensated proton leak. (A) Complex I-dependent and Complex II-dependent oxygen (O_2_) consumption in isolated forebrain mitochondria exposed to CoQ2 (100 μM). EtOH served as a vehicle control. Oligo, oligomycin; DNP, dinitrophenol. (B) Simultaneous measurement of O_2_ consumption and mitochondrial membrane potential (ΔΨm) during leak respiration in isolated forebrain mitochondria (mito) exposed to CoQ2 (100 μM). Representative traces of O_2_ consumption (red) above with ΔΨm (blue) below. Numbers are O_2_ consumption rates (nmol•min^−1^•mg mitochondrial protein^−1^). ΔΨm was measured following tetraphenylphosphonium ion (TPP^+^) calibration. (C) Steady-state ETC complex kinetic activities in unexposed isolated forebrain mitochondria or mitochondria exposed to CoQ2 or EtOH. First-order rate constants expressed as turnover number (TN) were determined for Complexes III and IV. Data are means ± SD. For (A and C) n = 4–6. P values were calculated by one-way ANOVA. *p < 0.05, **p < 0.01, ***p < 0.001, ****p < 0.0001.

Next, we exposed isolated mouse forebrain mitochondria to CoQ2 *in vitro* to determine how it affects the ability of mitochondria to generate and maintain ΔΨm. First, we measured oxygen consumption in actively respiring forebrain mitochondria using polarography. The control vehicle, ethanol, had no significant effect on oxygen consumption (Fig. 2A). In contrast, CoQ2 significantly decreased Complex I-dependent state 3 respiration and significantly reduced maximal oxygen consumption induced by dinitrophenol (state 3_DNP_) independent of substrate (Fig. 2A). On the other hand, CoQ2 significantly increased Complex II-dependent state 4 and oligomycin-induced state 4 respiration (Fig. 2A). Thus, CoQ2 inhibited electron transport and stimulated Complex II-dependent proton leak. Next, we simultaneously measured oxygen consumption and ΔΨm during Complex II-dependent leak respiration to determine the effect of CoQ2 on ΔΨm. CoQ2 immediately increased leak respiration as evidenced by a sudden increase in the rate of oxygen consumption and caused a concomitant and precipitous decline in ΔΨm (Fig. 2B). Thus, CoQ2 induced uncompensated proton leak, preventing mitochondria from generating and maintaining an adequate ΔΨm.

**Fig. 3.**
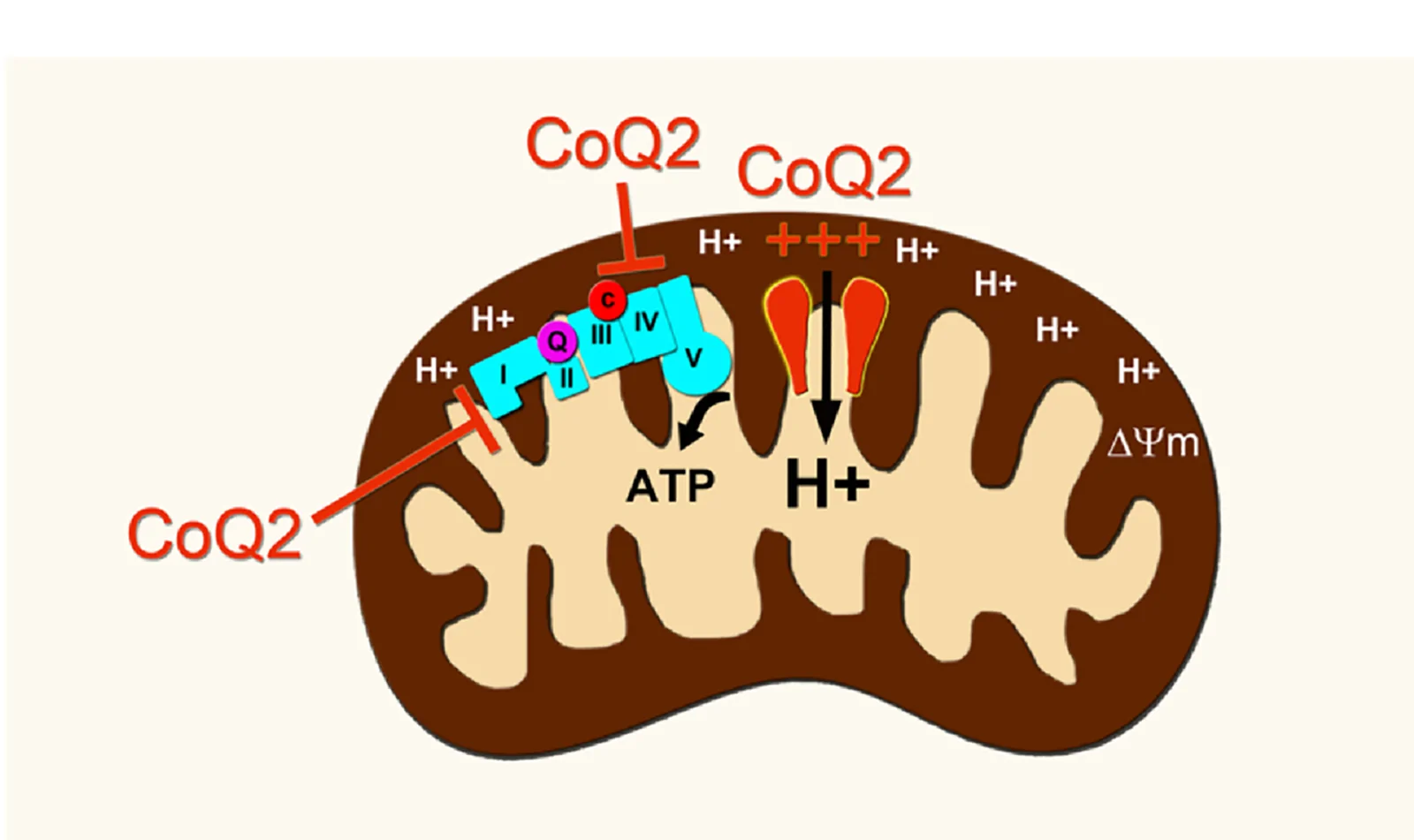
Mechanism by which coenzyme Q2 compromises the mitochondrial membrane potential. CoQ2 induces excessive proton leak and simultaneously inhibits Complexes I and IV within the ETC. The combined effects dissipate ΔΨm while preventing a compensatory increase in substrate oxidation to restore or maintain the proton motive force.

Next, we quantified the kinetic activity of each ETC enzyme complex during exposure to CoQ2 or ethanol vehicle in isolated mitochondria to determine where CoQ2 interfered with the ΔΨm-generating capacity. Ethanol had no effect on any ETC enzyme complex and CoQ2 had no significant effect on Complex V (Fig. 2C). In contrast, CoQ2 significantly inhibited steady-state Complex I activity, linked Complex I+III activity, and Complex IV activity in a concentration-independent manner (Fig. 2C). In addition, the highest concentration of CoQ2 also inhibited steady-state Complex III activity (Fig. 2C). Both CoQ2 concentrations stimulated linked Complex II+III activity and the highest concentration increased steady-state Complex II activity (Fig. 2C). However, the combined inhibitory effects on Complexes I and IV best explain the disruption in ΔΨm-generating capacity in the setting of CoQ2-induced excessive proton leak. Thus, as with Ub5 (CoQ1), CoQ2 caused a precipitous decline in ΔΨm in forebrain mitochondria due to excessive mitochondrial proton leak combined with ETC inhibition (Fig. 3) [11].

### Blockade of coenzyme Q2-induced proton leak

**Fig. 4.**
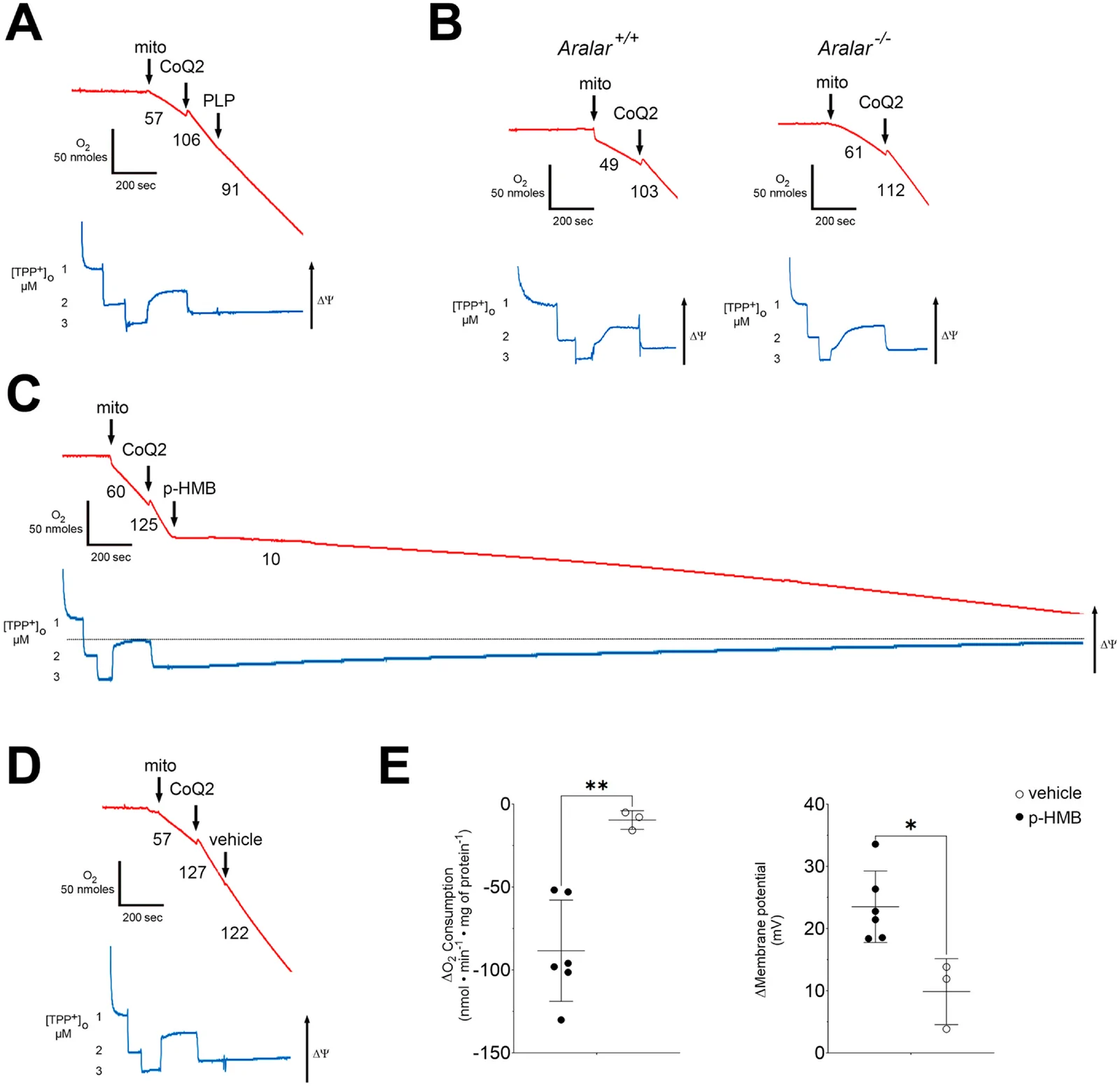
Source of coenzyme Q2-induced proton leak. Oxygen consumption and mitochondrial membrane potential (ΔΨm) were simultaneously measured during leak respiration in isolated forebrain mitochondria exposed to CoQ2 (100 μM). Various inhibitors were used to determine source of leak. Representative traces of O_2_ consumption above with ΔΨm (blue) below in mitochondria (mito) exposed to (A) pyridoxal 5’-phosphate (PLP), (C) hydroxymercuribenzoate (p-HMB), or (D) equal volume vehicle. Representative traces in uninhibited mitochondria from (B) *Aralar*^*+/+*^ or *Aralar*^*−/−*^ mouse forebrain are depicted. Numbers are O_2_ consumption rates (nmol•min^−1^•mg mitochondrial protein^−1^). ΔΨm was measured following tetraphenylphosphonium ion (TPP^+^) calibration. Inhibition of leak was identified in (C) as a decrease in O_2_ consumption with a concomitant increase in ΔΨm (returned to baseline (dashed line) after several minutes). **(E)** ΔO_2_ consumption and Δmembrane potential during CoQ2 leak were quantified following the addition of p-HMB or vehicle. Data are means ± SD. N = 3–6 per group. P values were calculated by paired Student’s t-test. *p < 0.05, **p < 0.01.

Subsequently, we attempted to identify the major source of CoQ2-mediated leak. To do so, we screened several specific and non-specific inhibitors for the ability to block such proton leak in isolated forebrain mitochondria. We assessed for a decline in oxygen consumption with a concomitant rise in ΔΨm during leak respiration as evidence of successful blockade. Previous work identified the aspartate-glutamate carrier, Aralar, as a functional anesthetic target and source of Ub5 (CoQ1)-mediated proton leak [11]. So, first we tested the non-specific inhibitor, pyridoxal 5’-phosphate (PLP), to determine the role of Aralar in CoQ2-mediated leak. Surprisingly, PLP had little to no effect (Fig. 4A). To confirm this finding, we then assessed the effect of CoQ2 on leak respiration in *Aralar* knockout mouse forebrain mitochondria along with wild-type littermate controls. CoQ2 caused a similar increase in the rate of oxygen consumption and precipitous decline in ΔΨm in both strains, indicating that, unlike Ub5 (CoQ1), CoQ2-induced leak is not mediated by Aralar (Fig. 4B).

Next, we tested specific inhibitors of the adenine nucleotide translocase (ANT), uncoupling proteins (UCPs), and the mitochondrial permeability transition pore (mPTP). These inhibitors minimally affected the rate of leak respiration and ΔΨm during CoQ2 exposure, indicating that the ANT, UCPs, and mPTP were not major source(s) of CoQ2-mediated leak (Supplemental figure). So, then we tested the non-specific inhibitor, p-hydroxymercuribenzoate (p-HMB). The rate of oxygen consumption decreased immediately following addition of p-HMB and ΔΨm recovered to pre-CoQ2 levels following a slow and steady rise (Fig. 4C). These effects indicated that p-HMB blocked the major source of CoQ2-induced leak. The changes in the rate of oxygen consumption and ΔΨm following p-HMB were both significantly different from vehicle-exposed values (Figs. 4D, 4E). However, despite the ability to successfully inhibit CoQ2-mediated leak, we were unable to identify the exact source given the non-specific nature of p-HMB inhibition.

## Discussion

Here we found that the short chain ubiquinone analog, CoQ2, acts as an anesthetic agent. This is the second molecule in the quinone class of compounds to demonstrate such a property. Thus, benzoquinones represent a sub-class of anesthetics. As with Ub5 (CoQ1), CoQ2 induced brief and reversible loss of wakefulness in mice shortly after injection [11]. However, we observed some key differences from Ub5 (CoQ1)-mediated hypnosis. First, the ED_50_ for CoQ2 was ~20 % higher than that of Ub5 (CoQ1)(~100 mg/kg for CoQ2 and ~80 mg/kg for Ub5 (CoQ1)) [11]. Second, the duration of CoQ2-induced behavioral quiescence was relatively longer than what was previously reported for Ub5 (CoQ1) [11]. Finally, loss of righting was delayed following CoQ2 injection, unlike the immediate onset of LORR after injection of Ub5 (CoQ1) [11].

Each of these distinct features could relate to the size and molecular weight of CoQ2 (~20 % greater than Ub5 (CoQ1)). However, another possible explanation is the relatively longer CoQ2 isoprenoid tail given that both molecules share an identical benzoquinone head [13]. Ub5 (CoQ1) has a single isoprene tail unit, allowing it to rapidly diffuse across membranes to penetrate the CNS and gain access to neuronal mitochondria [12]. On the other hand, CoQ2 has two isoprene tail units and is relatively more hydrophobic [13]. Although CoQ2 is thought to be able to readily diffuse across membranes, our findings suggest that it takes longer to traverse the blood-brain barrier than Ub5 (CoQ1) [32]. It is not clear if greater hydrophobicity prolongs the diffusion process or if CNS uptake of CoQ2 relies, to some degree, on lipid trafficking mechanisms [32]. Since we did not measure brain levels of CoQ2 or assess how CoQ2 crosses the blood-brain barrier, we don’t have a definitive explanation at this point. So, future work will need to define exactly how CoQ2 gains access to the CNS.

Importantly, we found that CoQ2 and Ub5 (CoQ1) share similar biological activity within forebrain mitochondria [11]. Specifically, both CoQ analogs compromise ΔΨm by inducing excessive mitochondrial proton leak in combination with inhibition of the ETC [11]. Disruption of electron transport occurs at the level of Complex I and Complex IV for each analog, suggesting that enzyme inhibition probably relates to the benzoquinone head and not the side chain. In support of this, we previously found that CoQ0 (benzoquinone ring lacking a side chain) inhibits electron transport in actively respiring mitochondria [11]. The binding site for the CoQ head group has been defined within Complex I, thus the benzoquinone ring may be capable of mediating Complex I inhibition [33,34]. Prior work found that CoQ2 can inhibit Complex I activity and increase state 4 leak respiration in both heart and liver mitochondria as well [35]. Therefore, such biological activity may be generalizable. However, CoQ2 differentially affects mPTP opening, reactive oxygen species production, and apoptosis in a tissue- and cell-specific manner [35,36]. Thus, follow up work will need to study the effects of CoQ2 on oxidative stress and programmed cell death within the CNS. Although quinones have an intimate relationship with Complex I, it is unknown how they interact with Complex IV. As a limitation, our work did not assess the mechanism of enzyme inhibition. So, further investigation will be necessary to elucidate exactly how quinone analogs inhibit both enzyme complexes.

With regard to proton leak, we previously established that Ub5 (CoQ1) directly activates the aspartate-glutamate carrier, Aralar, to compromise ΔΨm [11]. However, this was not the case with CoQ2. Although we were unable to definitively identify the source of CoQ2-mediated leak, we determined that it could be effectively blocked by p-HMB. p-HMB is known to inhibit several members of the mitochondrial SLC25 carrier family or their yeast orthologs in a non-specific manner [23–29]. These include SLC25A33 (a mitochondrial transporter of pyrimidine nucleotides), the mitochondrial dicarboxylate carrier (SLC25A10), the mitochondrial adenyl nucleotide antiporter (SLC25A24), mitochondrial glutamate carrier 1 (GC1)(SLC25A22) and GC2 (SLC25A18), mitochondrial ornithine transporter 1 (SLC25A15) and ornithine transporter 2 (SLC25A2), the mitochondrial glycine transporter (SLC25A38), and the mitochondrial phosphate carrier (PiC)(SLC25A3) [23–29].

Of these transporters and carrier proteins, only GC1, GC2, ornithine transporter 1, and the PiC have the ability to cause leak by importing a proton into the mitochondrial matrix [26,37]. The PiC remains as the sole candidate of interest in this group when considering that fact that GC1, GC2, and ornithine transporter 1 can be inhibited by PLP (which failed to affect CoQ2-induced leak) [26,38]. Interestingly, the PiC was previously identified as a binding target of the intravenous anesthetic, propofol [39]. So, it may be a pharmacological target of a variety of sedative-hypnotic agents including CoQ2. This will need to be tested going forward.

For obvious reasons, we were unable to assess the source of CoQ2-induced proton leak (the PiC or otherwise) as a functional anesthetic target as part of this study. This prevented us from demonstrating a causal link between the precipitous decline in ΔΨm and CoQ2-induced loss of wakefulness. Therefore, future work will need to do so, utilizing specific pharmacological blockade and genetic silencing approaches. Despite these limitations, we can definitively conclude that CoQ2 and Ub5 (CoQ1) cause excessive proton leak via different mechanisms within mitochondria. It is likely that disparities between these two agents relate to the length of their isoprenoid tail given the structural similarities and differences between molecules. Although it is unknown how each CoQ analog interacts with its respective leak source, it is plausible that the hydrophobic tail attached to the benzoquinone ring is the key structural feature that elicits proton leak. Such a notion will need to be investigated in follow-up studies. Exploring the effects of other quinone analogs with longer side chains will help to answer these questions and aid in further characterization of this sub-class of anesthetics. Ultimately, a better understanding of how benzoquinones perturb mitochondrial biology will help to more precisely define exactly how mitochondria mediate the anesthetic response.

## Conclusion

CoQ2 has sedative-hypnotic properties, confirming that benzoquinones represent a sub-class of anesthetics. Although CoQ2 induces unconsciousness and compromises ΔΨm within forebrain mitochondria in a similar manner as the anesthetic, Ub5 (CoQ1), there are key differences between analogs. The comparable effects likely relate to the identical benzoquinone head group shared between molecules while the disparities in anesthetic response and mitochondrial leak are likely due to the length of the isoprenoid tail.

## Supplementary Material

supplementary figure

## Data Availability

The authors declare that all data supporting the findings are available within the manuscript and its Supplemental Material.

## Appendix A. Supporting information

Supplementary data associated with this article can be found in the online version at doi:10.1016/j.cmp.2025.12.001.
