# Supplementary material for: Sedative-hypnotic properties and mitochondrial effects of coenzyme Q2 in mice
Source: Curr Mol Pharmacol. Author manuscript; Available in PMC 2026 Jun 16. (PMC13271189; doi:10.1016/j.cmp.2025.12.001)

**Supplemental figure. Effect of specific inhibitors on coenzyme Q2-induced proton leak.** Various inhibitors were used to determine source of leak. O**xygen (O_2_) consumption and mitochondrial membrane potential (**ΔΨm) were **simultaneously measured during leak respiration in isolated forebrain mitochondria (mito) exposed to CoQ2 (100 µM). (A)** Carboxyatractyloside (cAT), **(B)** cyclosporine (CsA), and **(C)** guanosine diphosphate (GDP) were added to specifically inhibit the adenine nucleotide translocase, the mitochondrial permeability transition pore, and uncoupling proteins**. Representative traces of O_2_ consumption above (red) with** ΔΨm **(blue) below are depicted. Numbers are O_2_ consumption rates** (nmol•min^-1^•mg mitochondrial protein^-1^**).** ΔΨm was measured following tetraphenylphosphonium ion (TPP^+^) calibration. *n* = 3-5 biological replicates from 3-5 different mice.


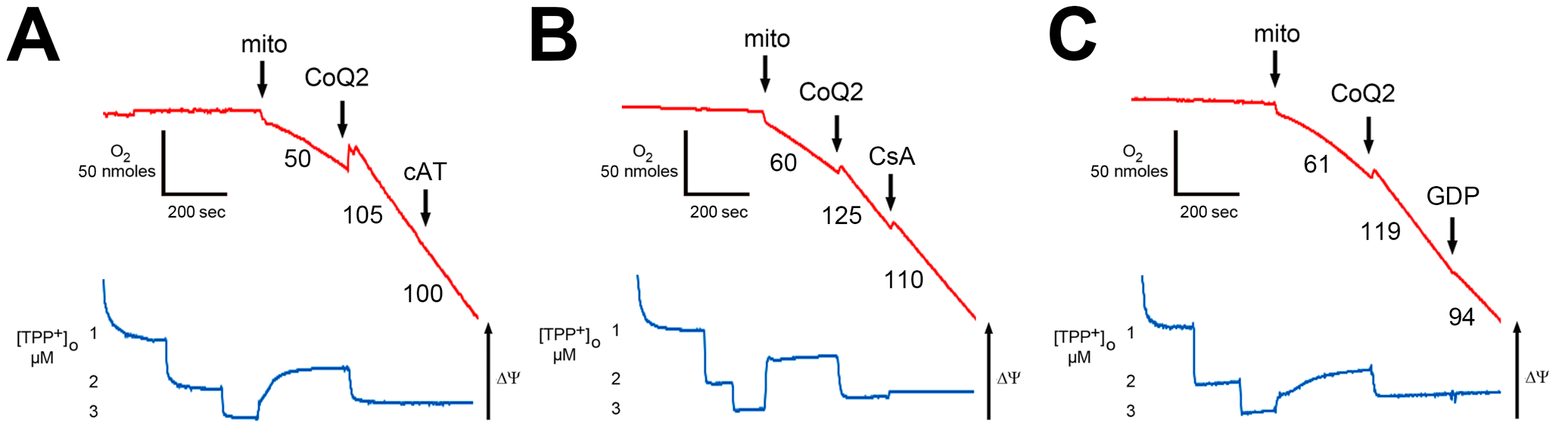

Supplement: supplementary figure [file NIHMS2181243-supplement-supplementary_figure.docx]
